# Is double-J stent mandatory in complete supine percutaneous nephrolithotomy for adult patients with staghorn renal stones?

**DOI:** 10.1186/s12894-024-01610-9

**Published:** 2024-10-07

**Authors:** Siavash Falahatkar, Samaneh Esmaeili, Samira Kazemi, Fatemeh Sheikhi, Hosna Norouzi

**Affiliations:** Urology Research Center, School of Medicine, Razi Hospital, Guilan University of Medical Sciences, Rasht, Iran

**Keywords:** Percutaneous nephrolithotomy (PCNL), Supine PCNL, Double-J stent, Staghorn stones, Ureteral catheter

## Abstract

**Background:**

It is controversial whether double-J (DJ) stent insertion is necessary in tubeless percutaneous nephrolithotomy (PCNL) for patients with staghorn stones. We compared the outcomes of using ureteral catheters and double-J stents in tubeless complete supine PCNL (csPCNL) of staghorn stones.

**Methods:**

In this analytical cross-sectional study, from May 2008 to August 2022, 123 patients who underwent tubeless csPCNL were assessed. Patients were divided into two groups by either tubeless csPCNL with DJ stent (Group I; *n* = 23) or totally tubeless just with perioperative ureteral stent (Group II; *n* = 100). Demographic characteristics, stone-related factors, perioperative and postoperative parameters were compared in groups.

**Results:**

Baseline characteristics were comparable in groups. The operative time in group I was significantly longer than group II (68.26 vs. 55.25 min, *P* = 0.05). However, the duration of hospitalization in Group I was shorter than the other group (1.81 vs. 2.37 days, *P* = 0.03). Stone free rate was notably higher in Group I (90.5% vs. 79.8.0%) with no statistically significant difference. No significant differences were found in major complications. Patients in Group II had a significantly shorter time to return to normal life (6.48 vs. 7.91 day; *P* = 0.043). Multivariable linear regression showed the preoperative creatinine level and stone size can affect the operative time (*P* = 0.02). In addition, stone number and underlying disease can affect the length of hospital stay (*P* = 0.007 & 0.030, respectively).

**Conclusion:**

Although not inserting a double J stent after csPCNL has acceptable results, because of higher residual rate in staghorn stone which cause more incidence of renal colic, longer time of hospital stay and return to normal life, inserting DJ stent is recommended.

## Background

Percutaneous nephrolithotomy (PCNL) is widely recognized as the most effective treatment for complex renal stones such as staghorn stones [1]. PCNL technique standardly involves the insertion of a nephrostomy tube for drainage; however, it may cause severe post-operative discomfort [2]. To eliminate nephrostomy tube irritation, tubeless PCNL was developed in which a ureteral catheter or a double-J stent replaces the nephrostomy tube for internal drainage [3]. Reducing pain, complications, and length of hospital stay are some of the benefits of tubeless PCNL [4–6]. Recently, the safety and efficacy of a new technique called totally tubeless PCNL using no tubes (no stent nor nephrostomy tube) are showed in selected patients [7].

Despite the feasibility of both tubeless and total tubeless PCNL in patients with complex and staghorn stones [4, 7–9], dealing with these stones remains a considerable challenge for endourologists. Additionally, they are associated with a higher incidence of perioperative and postoperative complications [10]. Telha et al. showed that tubeless PCNL using double-J (DJ) stent has lower complication rate and shorter length of hospital stay compared to tubeless PCNL with external ureteral catheter (EUC) [11]. Also, replacing DJ stent with EUC is not recommended in patients with large stone size or large residual stone fragments [12].

The advantages of performing PCNL in the supine compared to prone position have been proven in many previous studies [13, 14]. Also, there are several studies have conducted on tubeless supine PCNL [10, 14, 15] and it has been shown that stone evacuation is better and easier in the.

supine position compared to prone, and there will be fewer residual stones, which causes less.

trouble for patients and decreased renal pelvis pressures [16]. but there is no study comparing outcomes of tubeless and totally tubeless PCNL in complete supine position. Therefore, in this study we aimed to compare the outcomes of tubeless complete supine PCNL (csPCNL) with insertion of DJ stent and totally tubeless csPCNL with perioperative ureteral stent.

## Materials and methods

### Study populations and design

**Fig. 1:**
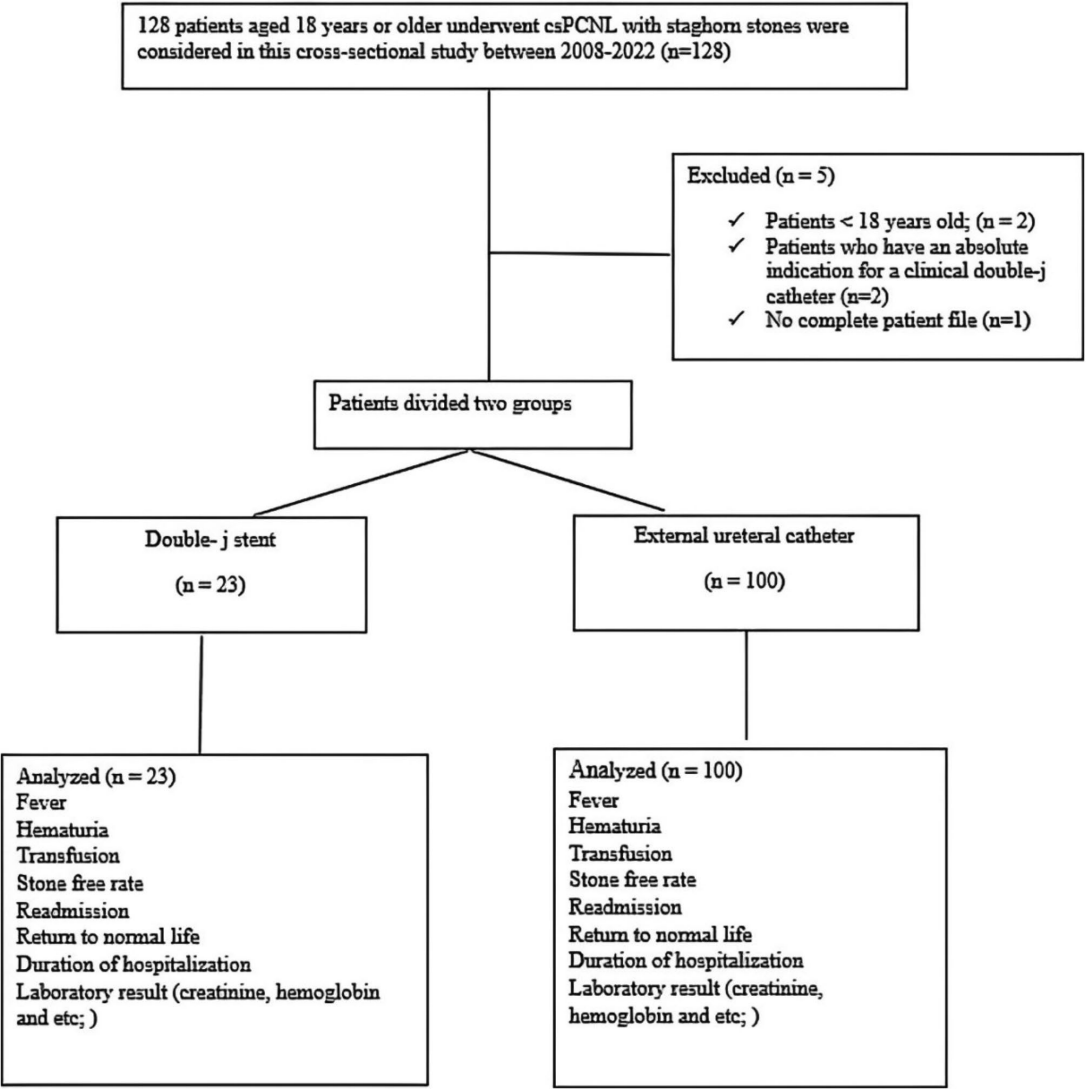
STROBE flow chart of eligible patients

After obtaining the ethics approval (IR.GUMS.REC.1402.040), in this cross-sectional study, between May 2008 and August 2022, 123 patients, over 18 years old, with staghorn stones who underwent csPCNL were assessed. Patients with absolute indications for a clinical DJ stent, single kidney, and ureteric perforation were excluded (Fig. 1).

Study cases were assigned into two groups according to stenting technique: Group I: tubeless csPCNL with DJ stent (*n* = 23) and Group II: totally tubeless csPCNL with perioperative ureteral stent (*N* = 100). Preoperative parameters and postoperative outcomes were recorded for all patients.

### Surgical procedure

**Fig. 2:**
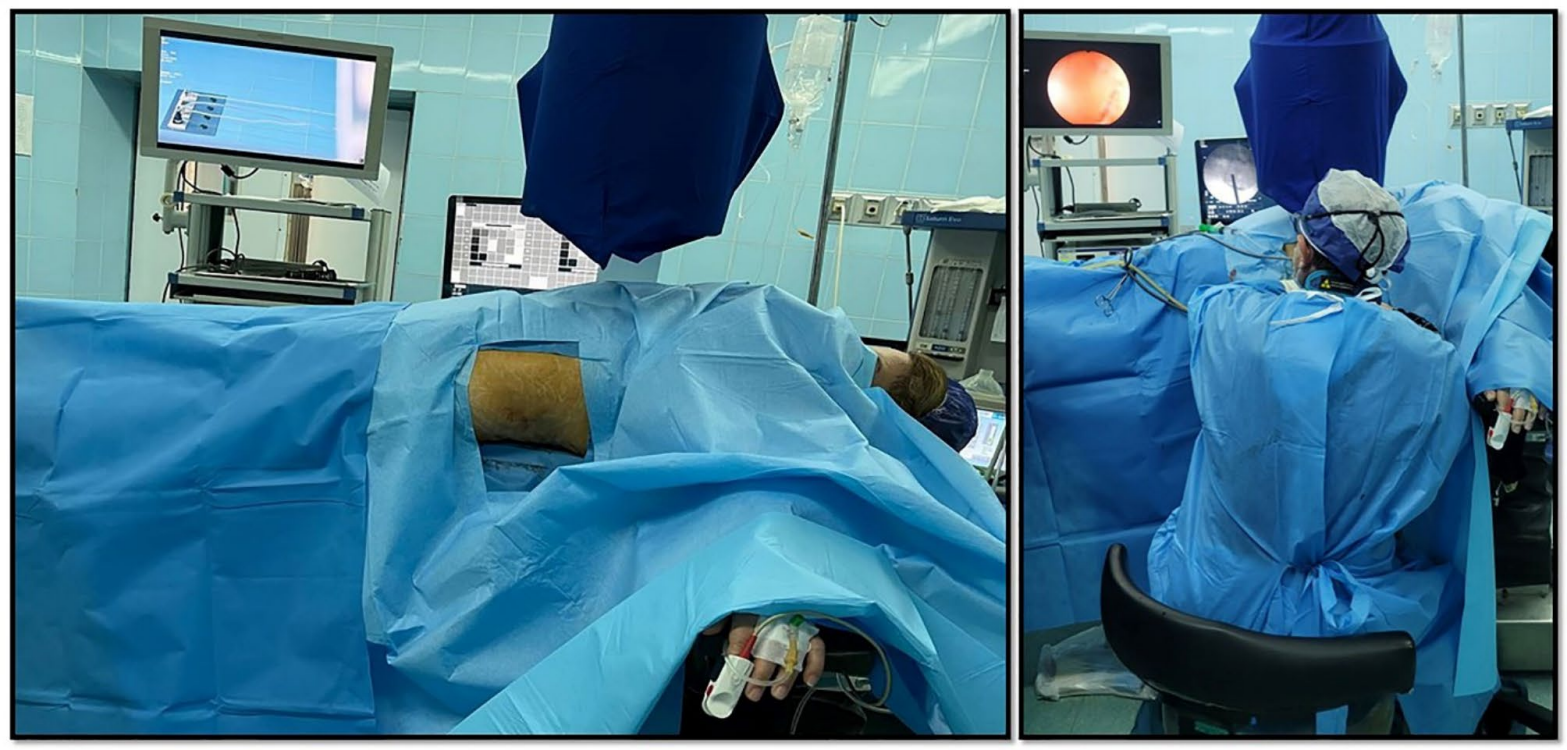
**left**: Patient underwent PCNL in complete supine position; **right**: Surgeon in sitting position during csPCNL

A single experienced urologist performed all csPCNL procedures under general or spinal anesthesia. Patients underwent PCNL in complete supine position, without elevating patients’ flanks or moving their legs (Fig. 2).

After catheterization, the patients were moved to the edge of the bed. Fluoroscopy guided the puncturing of the posterior subcostal with an 18-gauge needle; fluoroscopy guided the posterior subcostal puncture. The stone lithotripsy process employed pneumatic devices, utilizing a standard one-shot dilation method with a 28 or 30 Fr Amplatz sheet. All patients received perioperative prophylactic antibiotics. In Group I, DJ stent inserted without the use of a nephrostomy tube, and removed it two to four weeks after surgery. Patients in Group II did not receive a DJ stent or nephrostomy tube after csPCNL. Instead, a ureteral stent was placed and removed immediately after operation or on the day of discharge. The decision to insert or not to insert a double J stent was made based on the surgeon’s preference according to the patient’s condition, perioperative complications and the presence of suspected residual stones.

### Evaluations

The operation time was calculated from the point of puncture skin with needle to the point of suturing (skin to skin). Ultrasonography and/or KUB were used at the 4th postoperative week to check surgical success, which was defined as the absence of stones or fragments ≤ 4 mm. All demographic characteristics, stone related factors, laboratory parameters, pre-operative, intra-operative, and post-operative parameters were recorded for all patients and compared between two groups.

### Statistical analysis

We analyzed the acquired data using statistical data science software (SPSS) version 26. The normality of the data was analyzed by using the Kolmogorov-Smirnov test. The data are expressed as the mean (± standard deviation) and number (percentage), depending on the type of data. To determine the comparison of outcomes between two independent groups (DJ stent and EUC), if the data are qualitatively nominal (categorical data), we used the chi-square or Fisher’s test. If the shape of distribution or Kolmogorov-Smirnov tests in the study’s quantitative data (age, BMI, etc.) did not show normality in two groups, we used the Maan Witney U test. We used multivariable linear regression to predict factors affecting outcomes (csPCNL). A P-value < 0.05 was considered statistically significant. In our study, the missing data less than 5%; therefore, complete-case analysis (CCA) methods were used.

## Results

Patient demographics, preoperative clinical characteristics, and stone-related factors were comparable between the two groups except in creatinine level. Patients in Group I had significantly higher level of creatinine (Cr) than another group (*P =* 0.002) (Table 1).

**Table 1:** Baseline characteristics of patients in two groups

Variable	Group 1 (*N* = 23)	Group 2 (*N* = 100)	*P*-value
**Sex (%)**			0.85
Male	12 (52.2)	50 (50.0)	
Female	11 (47.8)	50 (50.0)	
**Age (years**,** mean ± SD)**	55.09 ± 11.87	50.87 ± 12.45	0.13
**BMI (kg/m**^**2**^, **mean ± SD)**	29.68 ± 6.34	28.24 ± 4.49	0.20
**Hypertension**	10 (45.5)	37 (38.1)	0.52
**Diabetes mellitus**	6 (27.3)	26 (27.7)	0.97
**Ischemic heart disease**	1 (4.8)	10 (10.8)	0.40
**Hypothyroidism**	0	7 (7.0)	0.19
**Hyperlimidia**	5(21.7)	11(11.7)	0.16
**Preoperative Hb (g/dl**,** mean ± SD)**	12.80 ± 2.17	13.15 ± 1.74	0.46
**Preoperative Cr (mg/dl**,** IQR)**	1.28 (1.10–1.70)	1.00 (0.90–1.20)	0.002
**Stone burden (mm**,** mean ± SD)**	44.67 ± 25.06	44.98 ± 18.38	0.95
**History of stone surgery (%)**	12 (52.2)	42 (42.0)	0.30
**Stone side (%)**			0.52
Left	9 (40.9)	46 (48.4)	
Right	13 (59.1)	49 (51.6)	
**Stone number (%)**			1.00
Single	7 (33.3)	30 (33.3)	
Multiple	14 (60.9)	60 (66.7)	
**Type of Staghorn (%)**			0.73
Partial	12 (52.2)	55 (58.5)	
Complete	11 (47.8)	39 (41.5)	
**Hydronephrosis (%)**			0.12
None or mild	15(65.2)	47(47.5)	
Moderete or Severe	8(34.8)	52(52.5)	
**Anesthesis (%)**			1.00
General	23(100)	98(98.0)	
Spinal	0	2(2.0)	

BMI: body mass index; Hb: Hemoglobin; Cr: creatinine

Continuous variables were compared by independent t- test or Mann-Witney U test. Qualitative variables were compared by chi-square or fisher exact test

The mean operative time was longer in Group I, and there was a slightly significant difference between the two groups (*P* = 0.055). The standardized mean difference in operative time between the two groups was equal to -0.54, which indicated a medium effect (SMD d cohen = -0.54; 95% confidence interval: -0.08 to -0.99). Although there was no statistically significant difference between two groups regarding the stone free rate, the success rate of the operation was notably higher in group 1 than group II from a clinical point of view (90.5% vs. 79.8%). The incidence of major complications was higher in Group II with no significant difference. Consequently, the postoperative hospitalization period in Group II was significantly longer than Group I (2.37 ± 1.37 vs. 1.81 ± 0.95 days; *P* = 0.03). Patients in Group II were returned to their normal life (activity) after 7.91 days which was significantly longer than this period in Group 1 (6.48 days) (*P* = 0.04). The main reason for this difference can be the complaint of patients in Group II of renal colic, which is caused by residual stones. Only 2 patients in Group II were readmitted to the hospital (Table 2).

**Table 2:** Perioperative and postoperative outcome of patients in two groups

Variable	Group 1 (*N* = 23)	Group 2 (*N* = 100)	*P*-value
**Mean operative time** (mins)	68.26 ± 29.17	55.25 ± 22.81	0.05
**Hb drop** (mean ± SD, g/dl)	1.20 ± 1.04	1.58 ± 1.38	0.15
**Cr change** (IQR, mg/dl)	0.10 (0.05–0.3)	0.10 (0-0.19)	0.53
**Complications** (%)	3 (13.0)	24 (24.0)	0.25
Fever	2 (9.1)	12 (12.5)	0.65
Hematuria	0 (0.0)	2 (2.0)	0.49
Blood transfusion	1 (5.0)	13 (15.5)	0.21
**Analgesic requirement** (mean ± SD, mg)	46.00 ± 44.62	45.70 ± 39.26	0.97
**Stone free rate (%)**	19(90.5%)	71(79.8%)	0.26
**Hospital Stay** (days, mean ± SD)	1.81 ± 0.95	2.37 ± 1.37	0.03
**Return to normal life** (day)	6.48 ± 2.35	7.91 ± 3.15	0.04
**Readmission to hospital** (%)	0 (0.0)	2 (2.0)	0.49

Hb: Hemoglobin; Cr: creatinine

Continuous variables were compared by independent t- test or Mann-Witney U test. Qualitative variables were compared by chi-square or fisher exact test

Multivariable linear regression was performed to identify factors affecting the duration of operation, hospitalization and return to normal life. As shown in Table 3, the level of preoperative Cr and stone size can affect the operative time in double-j compared another group (*P* = 0.02).

**Table 3:** Linear regression analysis to identify the cofounders of affecting operative time

Predictors	Unstandardized B Coefficients	Standardized B Coefficients	Standard Error	95%CI	*P*-value	VIF
Age	-0.16	-0.08	0.22	-0.60 to 0.27	0.46	1.39
BMI	0.35	0.07	0.48	-0.61 to 1.32	0.47	1.08
Preoperative Cr	15.45	0.24	6.56	2.42 to 28.48	0.02	1.17
Stone number (multiple/ single)	4.93	0.09	4.96	-4.92 to14.79	0.32	1.02
Underlying disease (yes/no)	3.79	0.07	5.45	-7.04 to14.62	0.48	1.31
History of stone surgery (yes/no)	2.87	0.05	4.77	-6.60 to12.35	0.54	1.01
Stone size	0.28	0.22	0.12	0.04 to 0.53	0.02	1.04

Table 4 shows that the number of stones and underlying disease can affect the length of hospital stay.

**Table 4:** Linear regression analysis to identify the cofounders of affecting hospital stay

Predictors	Unstandardized B Coefficients	Standardized B Coefficients	Standard Error	95%CI	*P*-value	VIF
Age	0.01	0.16	0.01	-0.005 to 0.04	0.13	1.39
BMI	-0.04	-0.15	0.02	-0.09 to 0.01	0.12	1.08
Preoperative Cr	-0.07	-0.02	0.34	-0.75 to 0.61	0.83	1.19
Stone number (multiple/ single)	-0.72	-0.26	0.26	-1.25 to -0.20	0.007	1.02
Underlying disease (yes/no)	-0.62	-0.23	0.29	-1.20 to -0.04	0.03	1.34
History of stone surgery (yes/no)	-0.30	-0.11	0.25	-0.79 to 0.19	0.23	1.01
Stone size	-0.009	-0.13	0.007	-0.02 to 0.004	0.15	1.04

Finally, in linear regression analysis, none of the confounding variables had a significant effect on the time to return to normal life. Only the number of stones had a very slight effect on this factor. (Table 5)

**Table 5:** Linear regression analysis to identify the confounder affecting return to normal life

Predictors	Unstandardized B Coefficients	Standardized B Coefficients	Standard Error	95%CI	*P*-value	VIF
Age	0.01	0.05	0.02	-0.04-0.06	0.65	1.39
BMI	-0.01	-0.02	0.06	-0.13-0.10	0.77	1.08
Preoperative Cr	-0.36	-0.04	0.81	-1.97-1.24	0.65	1.17
Stone number (multiple/ single)	1.13	0.18	0.61	-0.08-2.35	0.06	1.02
Underlying disease (yes/no)	-0.52	-0.08	0.67	-1.86-0.81	0.43	1.31
History of stone surgery (yes/no)	0.83	0.13	0.59	-0.33-2.004	0.16	1.01
Stone size	0.004	0.02	0.01	-0.02-0.03	0.80	1.04

## Discussion

Although totally tubeless PCNL without a nephrostomy catheter, a double J stent, or a ureteral catheter has numerous benefits for patients and many studies have shown its safety and efficacy [2, 7, 17], there is still concern about the consequences of not inserting a stent for challenging complex stones. In addition, a major limitation of the published results is that the surgeon is not blinded in these studies and can decide whether to leave the stent or not. Therefore, this technique can be preferred only in selected cases.

In our study, comparing the outcomes of tubeless csPCNL using DJ stent with totally tubeless just with perioperative ureteral stent csPCNL showed acceptable and comparable results for both techniques. There were no significant differences between the two stenting techniques regarding the patients’ baseline characteristics.

In the present study, the mean operative time in Group II was shorter than another group with a DJ stent which consider slightly significant (*P* = 0.05). In pervious study, no significant difference was reported for mean operative time in groups with DJ stent and ureteral catheter [18–20]. It should be noted that none of these studies has been conducted staghorn stones.

Several factors can affect the operative time such as patient and stone-related factors and perioperative outcomes. In our study the level of preoperative Cr and stone size were found as predictive factors of operative time. These factors also encouraged the surgeon to insert DJ stent for patients.

In previous studies that have compared different stenting techniques, the success rate has been less discussed. In a small number of studies, no significant difference has been reported between the stone free rate in different stenting techniques [18, 19]. Similarly, in our study, no statistically significant difference was found between two groups regarding the stone free rate. However, the success rate was notably higher in Group I than Group II (90.5% vs. 79.8%), which is clinically remarkable.

In our study, patients with ureteral catheter had longer hospital stays than the other group (2.37 vs. 1.81). The duration of hospitalization is affected by many factors, which in this study, the number of stones and underlying diseases were identified as predictive factors.

Our findings are similar to a study by Telha et al. [11] that showed inserting DJ stent after tubeless PCNL can reduce the length of hospital stay. Also, in a study by Habib et al., longer hospital stay was reported in group with EUC than group with DJ stent (4.2 ± 1.71 vs. 3.9 ± 0.92), however this difference was not statistically considered significant [15]. Contrary to the results of our study, Gonen et al. [18] and a meta-analysis by Chen et al. [19] found no difference in length of hospital stay between patients undergoing tubeless PCNL with EUC and DJ stent.

In our study, the frequency of major complications, including fever, hematuria and blood transfusion in Group I and II were reported no statistically significant difference. Similar to our findings, previous studies [15, 18, 19] reported no significant difference between groups of patients underwent tubeless PCNL with DJ stents and EUC. However, Telha et al. [11] showed that Tubeless PCNL with DJ placement decrease the complications compared to EUC.

Our results also showed shorter time to return to normal life in patients with DJ stent compared to another group (6.48 vs. 7.91 days; *P* = 0.04). In multivariable linear regression, none of the confounding factors affected return to normal life. Only the number of stone had a slight impact on this factor (*P* = 0.06) However, we suspect that the main reason for the longer time to return to normal life in patients without DJ stents could be the experience of more frequent renal colic, which a large number of patients in Group II complained about. Although this problem did not cause the need to re-hospitalize and only two patients in this group were readmitted to the hospital.

So far, no comparison has been made between the two groups of patients undergoing tubeless PCNL in terms of the return to normal life, and only two studies compared this factor in two groups of patients underwent totally tubeless PCNL and standard PCNL (with a nephrostomy tube) (21–22).

However, regardless of position or lithotripsy method, DJ stent placement after PCNL in complex stone seems to be a safe way for stone particles or clot passage with less or even no pain and discomfort., but in group II; they experienced more renal colic due to residual stone, and this caused a longer time to return to normal life for them. We believed that the decision to insert or not to insert a DJ stent should be individual, and with accurate and correct selection of patients, the success rate can be increased and complications can be reduced.

The limitations of our study were its non-randomized design and small sample size. Despite this limitation, this study is first comparison of inserting DJ stent and ureteral catheter in tubeless csPCNL in patients with challenging staghorn stones.

## Conclusion

Although the need for DJ stent in PCNL for staghorn stone is challenging, inserting DJ stent is recommended in this group of patients because of shorter hospital stay, shorter time to return to normal life and better success rate.

## Data Availability

The datasets used and/or analyzed during the current study are available from the corresponding author on reasonable request.

## Abbreviations

csPCNL: Complete supine percutaneous nephrolithotomy
DJ: Double-J
EUC: External urethral catheter
KUB: Kidney, ureter and bladder
BMI: Body Mass Index
